# Supplementary material for: The interactome of intact mitochondria by cross-linking mass spectrometry provides evidence for coexisting respiratory supercomplexes
Source: Mol Cell Proteomics. 2017 Dec 8;17(2):216–32. doi: 10.1074/mcp.RA117.000470 (PMC5795388; doi:10.1074/mcp.RA117.000470)
Supplement: Supplemental Data [file supp_RA117.000470_133922_0_supp_23978_fzffwf.zip › spectra_annotation/mito_spectra_annotation/239-1-5-1-1-1.pdf]

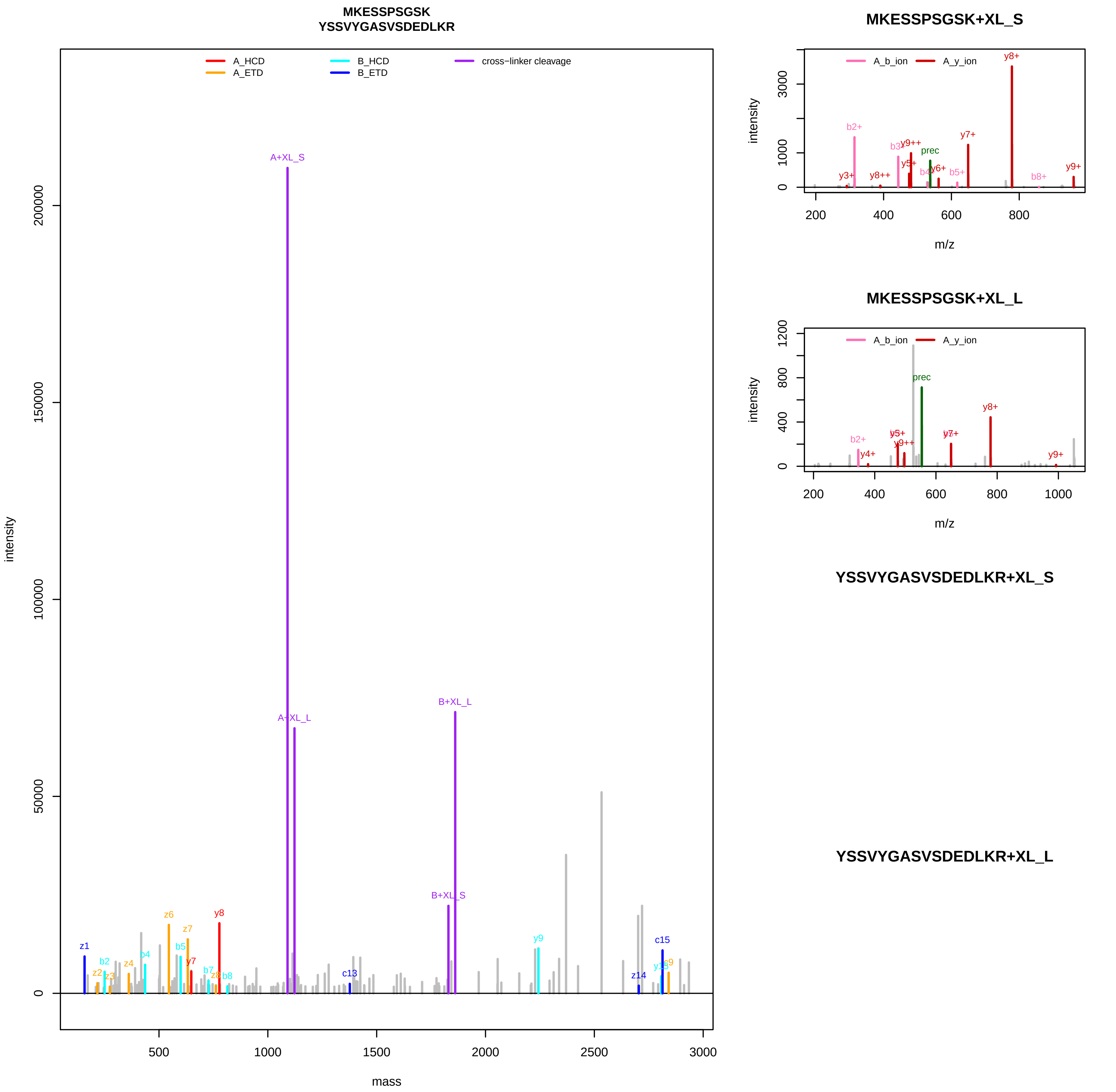
MKESSPSGSK
YSSVYGASVSDEDLKR
A_HCD
A_ETD
B_HCD
B_ETD
cross−linker cleavage
A+XL_S
A+XL_L
B+XL_L
B+XL_S
z1
b2
z2
z3
z4
b4
z6
b5
z7
y7
y8
b7
z8
b8
c13
y9
z14
c15
intensity
mass
0
50000
100000
150000
200000
500
1000
1500
2000
2500
3000
MKESSPSGSK+XL_S
A_b_ion
A_y_ion
y8+
b2+
y9++
b3+
y5+
prec
y7+
y6+
b4+
b5+
y3+
y8++
b8+
y9+
m/z
MKESSPSGSK+XL_L
y4+
y7+
YSSVYGASVSDEDLKR+XL_S
YSSVYGASVSDEDLKR+XL_L
